# Targeting Wnt/β-Catenin Pathway by Flavonoids: Implication for Cancer Therapeutics

**DOI:** 10.3390/nu15092088

**Published:** 2023-04-26

**Authors:** Pratibha Pandey, Fahad Khan, Sara A. Seifeldin, Khalid Alshaghdali, Samra Siddiqui, Mohamed Elfatih Abdelwadoud, Manish Vyas, Mohd Saeed, Avijit Mazumder, Amir Saeed

**Affiliations:** 1 Department of Biotechnology, Noida Institute of Engineering and Technology, Greater Noida 201306, India; shukla.pratibha1985@gmail.com; 2 Department of Medical Laboratory Science, College of Applied Medical Sciences, University of Ha’il, Hail 55476, Saudi Arabia; s.seifeldin@uoh.edu.sa (S.A.S.); k.alshaghdali@uoh.edu.sa (K.A.); 3 Medical and Diagnostic Research Centre, University of Hail, Ha’il 55473, Saudi Arabia; s.siddiqui@uoh.edu.sa; 4 Department of Public Health, College of Health Sciences, University of Ha’il, Hail 55476, Saudi Arabia; 5 Department of Histopathology and Cytology, Faculty of Medical Laboratory Sciences, University of Medical Sciences & Technology, Khartoum 11115, Sudan; mohdsanhori79@gmail.com; 6 School of Pharmaceutical Sciences, Lovely Professional University, Punjab 144411, India; manish.17410@lpu.co.in; 7 Department of Biology, College of Sciences, University of Hail, Ha’il 34464, Saudi Arabia; mo.saeed@uoh.edu.sa; 8 Department of Pharmacology, Noida Institute of Engineering and Technology (Pharmacy Institute), Greater Noida 201306, India; avijitmazum@yahoo.com; 9 Department of Medical Microbiology, Faculty of Medical Laboratory Sciences, University of Medical Sciences & Technology, Khartoum 11115, Sudan

**Keywords:** flavonoids, Wnt/β-catenin signaling, natural product, anticancer

## Abstract

The Wnt pathway has been recognized for its crucial role in human development and homeostasis, but its dysregulation has also been linked to several disorders, including cancer. Wnt signaling is crucial for the development and metastasis of several kinds of cancer. Moreover, members of the Wnt pathway have been proven to be effective biomarkers and promising cancer therapeutic targets. Abnormal stimulation of the Wnt signaling pathway has been linked to the initiation and advancement of cancer in both clinical research and in vitro investigations. A reduction in cancer incidence rate and an improvement in survival may result from targeting the Wnt/β-catenin pathway. As a result, blocking this pathway has been the focus of cancer research, and several candidates that can be targeted are currently being developed. Flavonoids derived from plants exhibit growth inhibitory, apoptotic, anti-angiogenic, and anti-migratory effects against various malignancies. Moreover, flavonoids influence different signaling pathways, including Wnt, to exert their anticancer effects. In this review, we comprehensively evaluate the influence of flavonoids on cancer development and metastasis by focusing on the Wnt/β-catenin signaling pathway, and we provide evidence of their impact on a number of molecular targets. Overall, this review will enhance our understanding of these natural products as Wnt pathway modulators.

## 1. Introduction

Despite major advancements in cancer treatment and remission rates, many challenges remain in cancer management. Resistance to therapy, which is linked to disease recurrence and metastasis, still poses serious issues that require attention. Cancer is one of the main ailments that places a burden on the world’s healthcare systems. There were approximately 19 million new cases of cancer in 2020, and approximately 10 million people died from cancer worldwide [1]. There is an imperative requirement for coordinated efforts to find and describe more effective treatments, considering the devastating loss of human life. Wingless-type (Wnt)/β-catenin signaling is a crucial signaling pathway that has a substantial impact on the initiation and progression of many disorders, especially cancer. This evolutionarily conserved signaling cascade plays a critical role in embryonic development and maintains adult stem cell homeostasis and tissue regeneration [2,3]. According to pre-clinical and clinical findings, stimulation of the Wnt/β-catenin signaling cascade has been linked to disease pathogenesis, including carcinogenesis. The Wnt/β-catenin signaling pathway is aberrantly stimulated due to mutations in its constituent parts, which promote the migration and invasion of malignancy by increasing the expression of primary targets (c-myc and cyclin D1) that are governed by this pathway and are responsible for cellular growth and programmed cell death [4,5]. A link between cytoplasm and nucleus can be made via β-catenin, which is situated downstream of the Wnt/β-catenin signaling pathway. The expression level of a primary target is induced when β-catenin interplays with transcription factors, such as the T-cell factor (TCF)/lymphoid enhancer factor (LEF) family, or transcription cAMP-response element-binding protein (CREB)-binding protein (CBP)/p300 [6]. Due to the distinct locality of β-catenin in the Wnt/β-catenin signaling pathway, alterations in the upstream elements (Wnt ligand, Axin, and APC) of this pathway typically lead to aberrant accumulation of β-catenin and dysregulation of the signaling cascade. Hence, it is important in the management of different malignancies related to this signaling pathway that β-catenin inhibitors can shut off the abnormal stimulation of the Wnt/β-catenin signaling pathway induced by upstream signal alterations [7]. The identification of therapeutic drugs that can target and modify the function of signaling molecules in order to battle cancer has been sparked by the complexity of cell signaling in cancer. In order to achieve this, modulating the Wnt/β-catenin signaling pathway appears to be a promising treatment strategy, particularly when relying on the chemopreventive properties of natural products [8]. The adverse relationship between a diet enriched in fruits and vegetables and cancer incidence or progression has been noted in various epidemiological research studies [9,10]. In this context, recent studies have placed a lot of emphasis on flavonoids as possible therapeutics for Wnt/β-catenin-targeted cancers. Flavonoids are a class of phenolic chemicals found in plants, fruits, and vegetables and are distinguished by a carbon skeleton base of C6-C3-C6. Having already been identified as antioxidant, antidiabetic, anti-inflammatory, antibacterial, antiviral, and antitumor agents, this group of phytochemicals is commonly consumed in human diet and has attracted significant scientific interest [11,12,13,14]. Epigallocatechin-3-gallate, apigenin, genistein, luteolin, quercetin, silibinin, kaempferol, and naringenin have been reported as effective flavonoid-based anticancer therapies in recent years [15]. These flavonoids show potent therapeutic potential against various cancers through their inactivation of carcinogens, stimulation of cytotoxic activity, improved antioxidant activity, reduced angiogenesis, pro-apoptosis, suppression of cellular growth, and modulation of aberrant cell signaling pathways [16,17]. Although the mechanisms that correlate with the anticancer ability of flavonoids have been identified, the exact mechanism of action of each compound is still not completely understood. Thus, research is ongoing to completely understand the mechanisms underlying the anticancer action of different flavonoids in an effort to increase their effectiveness and reduce their drawbacks. For instance, the two primary modes of action, which are found in the majority of flavonoids, are a decrease in reactive oxygen species (ROS) and the triggering of apoptosis via death receptors [18]. However, recent studies have suggested that flavonoid-mediated targeting of abnormal cell signaling pathways could be a promising strategy for cancer management. In this context, the present review highlights the anticancer potential of flavonoids that modulate Wnt/β-catenin signaling in several malignancies. In addition, we briefly describe the associated anticancer mechanisms and cellular/molecular targets of the Wnt/β-catenin signaling cascade, which are directly or indirectly affected by these natural compounds.

## 2. Wnt/β-Catenin Signaling Pathway in Tumor Progression

The early stages of cancer development are caused by an aberrant modulation of the transcription factor β-catenin, which is a crucial part of the Wnt/β-catenin signaling pathway [19,20,21,22]. Glycogen synthase kinase 3β (GSK3β) and casein kinase 1 (CK1) promote the phosphorylation of β-catenin within the destruction complex, enhancing its ubiquitination and leading to the ensuing proteasomal breakdown [23]. The interaction of secretory cysteine-rich glycoprotein ligands called Wnts with LRP-5/6 receptors and FZD receptors initiates the β-catenin-mediated signaling cascade. When Wnt ligands are present, the interaction of Wnt ligands and receptors on the cell surface results in the stimulation of Dishevelled (DVL), which causes the assembly of the complex (AXIN, GSK3β, CK1, and APC) at the receptor [24]. Thus, an increase in the amount of cytosolic β-catenin is facilitated by the phosphorylation and repression of GSK3β. Unphosphorylated β-catenin in the cytoplasm translocates to the nucleus and aggregates, binding to TCF/LEF and co-activators, such as Pygopus and Bcl-9, to activate Wnt target genes, including c-Myc, cyclin D1, and CDKN1A, thereby leading to the elevation of TCF/LEF target genes. Moreover, various control mechanisms have been found to govern the phosphorylation and ubiquitination of β-catenin by the destruction complex. Wnt proteins are prevented from secreting outside of cells by Notum, which removes palmitoleate from them. Dickkopf (DKK), by selective binding to LRP5/6 receptors, inhibits the commencement of Wnt protein-mediated signaling. In addition, Wnt protein-dependent signaling is blocked by secreted FZD-linked proteins (sFRPs) that interact with FZD receptors. Wnt inhibitory factor (WIF) also prevents the signaling cascade by directly interacting with Wnt proteins [25]. ZNRF3 and RNF43, which are transmembrane elements with E3 ubiquitin ligase activity, interact with FZD proteins [26]. To improve Wnt signaling at low concentrations of Wnt ligands, 7-transmembrane receptors, including LGR4, LGR5, and LGR6, interact with R-spondins (RSPO) with great affinity [27,28].

Moreover, Wnt/β-catenin signaling coordinates with a number of cell signaling pathways, including the PI3K/Akt pathway, nuclear factor kappa-B (NF-κB), Sonic Hedgehog, Notch, Hippo/YAP, and epidermal growth factor receptor (EGFR), all of which play a crucial role in the carcinogenesis process [29,30,31]. The angiogenic properties of cancerous cells may be aided by EGFR and β-catenin complexes [32,33]. Furthermore, it has been established that Hippo signaling blocks the phosphorylation of DVL, nuclear accretion of β-catenin, and expression of β-catenin and TCF-target genes within the Wnt/β-catenin signaling cascade [34,35]. In glioblastoma cells, stimulation of the Wnt/β-catenin pathway, coupled with the PI3K/AKT/GSK-3 pathway, contributes to the molecular foundation of temozolomide chemoresistance [36]. AKT kinase might also stimulate β-catenin activity. As a result, it has been demonstrated that the cross talk between the Wnt/β-catenin and PI3K-AKT pathways promotes carcinogenesis and confers resistance to chemotherapeutic agents [37].

Several malignancies, such as ovarian, colon, pancreatic, skin, and lung cancers, are primarily caused by mutations in the Wnt/β-catenin pathway [38,39,40,41]. Using data from the Cancer Genome Atlas (TCGA) project, Sanchez-Vega et al. conducted a detailed landscape investigation of the mechanisms and trends of somatic variations in important oncogenic signaling pathways in 33 kinds of cancer. According to their research, Wnt pathway mutations are the most diverse among the various types of cancer, with CTNNB1 (β-catenin), APC, and TCF7 being the most frequently altered genes [42].

**Figure 1:**
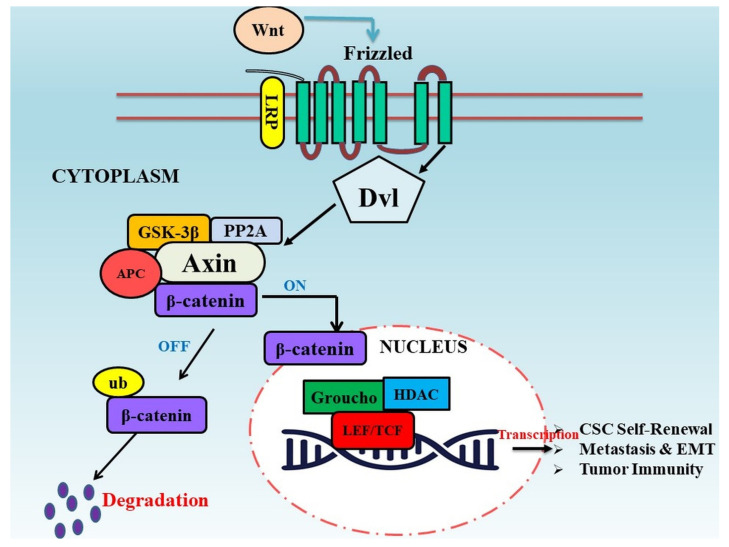
Wnt/β-catenin signaling pathway in tumor progression. The extracellular, cytoplasmic, and nuclear signaling events that constitute the Wnt/β-catenin signaling pathway can be therapeutically manipulated. β-catenin promotes the transcription of genes that are essential for CSCs, tumor metastasis, EMT, and tumor immunity after moving into the nucleus and interacting with TCF/LEF and a number of co-activators.

Even when Wnt ligands are absent, the Wnt/β-catenin pathway is still active in several cancerous cells [43]. APC mutations result in constitutively active nuclear β-catenin accumulation, an inactive degradation complex, and transcription of Wnt-dependent genes. Wnt signaling is also involved in the immunological response, tumor persistence, and metabolic changes that encourage and promote carcinogenesis [44] (Figure 1). A dysregulated Wnt/β-catenin signaling pathway promotes cancer stem cell regeneration, cellular proliferation, and metastasis, thus playing an important role in carcinogenesis and therapeutic responses [45,46]. The clinical application of medications that block Wnt/β-catenin signaling in cancer has been highlighted in several studies. The key elements of this pathway, including the Wnt ligand/receptor interface, β-catenin degradation complex, and TCF/β-catenin transcription complex, have been the focus of preclinical and clinical studies [47].

### 2.1. Correlation of Wnt Signaling in Cancer Stem Cells

The cancer stemness paradigm, which describes the capability of cancerous cells to self-renew, has been used to explain a variety of malignant characteristics [48]. The crucial significance of the Wnt/β-catenin pathway in the operation of normal and cancer stem cells is widely acknowledged, although the notion of cancer stemness is still widely debated [49]. The development and maintenance of leukemic stem cells and many other types of cancers have been linked to deregulated Wnt signaling [50]. The advancement of chronic-phase CML into the blast crisis stage as a result of GSK3β mutations and the stability of β-catenin in granulocyte–macrophage progenitor cells (GMP cells) are two examples of abnormal Wnt/β-catenin signaling in the formation of cancer stem cells [51]. A recent report suggested that relapses in patients are caused, at least to some extent, by stimulation of the Wnt/β-catenin pathway, irrespective of the repressive effect of tyrosine kinase inhibitors (TKI) on Wnt signaling in CML stem cells [52]. MiR29, which is involved in CD70 promoter methylation, is downregulated as a result of TKI treatment. The Wnt/β-catenin signaling pathway is activated by CD27, which is enhanced by CD70 overexpression [53]. Moreover, Wang et al. demonstrated that to produce AML leukemic stem cells from MLL-AF9-transduced progenitor cells, continuous stimulation of the canonical Wnt/β-catenin pathway is required [54]. The results of this study revealed that leukemic stem cells (LSC) may develop from more committed progenitor cells, in addition to hematopoietic stem cells (HSC), as a result of abnormal Wnt pathway activation. Giambra et al. recently showed that some small subpopulations of bulk T-cell acute lymphoblastic leukemia (T-ALL) highly stimulate the Wnt/β-catenin pathway. Leukemic stem cells were found to be significantly more prevalent in the GFP-positive Wnt-producing population than in the GFP-negative population, indicating that Wnt signaling is also necessary for T-ALL stem cell self-renewal. According to this concept, the transcription factor HIF1-α (Hypoxia-induced factor 1-alpha) appears to be accountable for promoting the transcription of β-catenin, and ablation of HIF1-alpha results in LSC targeting [55].

The epithelial-to-mesenchymal transition (EMT), another feature of cancer stem cells and metastasis, also seems to be significantly influenced by the Wnt signaling pathway [56,57,58]. The nuclear movement of β-catenin and the induction of canonical Wnt signaling are triggered by the decreased level of E-cadherin, which is typically firmly linked with β-catenin in healthy epithelium [59]. Moreover, the EMT marker gene, Slug, causes the nuclear movement of β-catenin [60]. Two potent EMT activators, Twist and Slug, are both potential targets for β-catenin [61]. In addition, a number of Wnt/β-catenin target genes, such as fibronectin, S100A4, CD44, L1CAM, MMP7, and uPAR, have been linked to invasion and migration [62].

### 2.2. Wnt Signaling and Metastasis

EMT, tumor neoangiogenesis, and tumor advancement are all aspects of metastasis, which develops from a primary tumor site and spreads to target tissues and organs in multiple steps. Malignant cells move across blood vessels to particular organs and tissues and invade them through infiltration, resulting in secondary tumor development [63,64]. Embryonic signaling may lead to the development of EMT in cancer stem cells (CSCs). Several transcriptional factors that promote EMT are upregulated as a result of Wnt, Hedgehog (Hh), transforming growth factor-β (TGF-β), or Notch activation. Through this process, tumor cells that are adherent, immobile, and epithelial in nature transform into mobile and invasive cells [65].

EMT is used to describe the conversion of polarized epithelial cells into migratory and invading mesenchymal cells [66,67]. SNAI2 is a transcriptional element that contributes to EMT. Phosphorylation of GSK3β by β-TrCP and concomitant ubiquitinylation by β-TrCP regulate the amount of SNAI2 in the cytoplasm. By preventing GSK3 kinase activity, the canonical Wnt/β-catenin signaling activation stabilizes SNAI2 and promotes EMT transcriptional pathways in cancer cells [68]. ASPP2 is another putative gene that controls EMT and binds to a complex of β-catenin and E-cadherin. This association prevents the N-terminal phosphorylation of β-catenin and subsequently stabilizes β-catenin. Decreased ASPP2 expression drives the EMT process and is related to poor prognosis in breast and hepatocellular carcinomas [69]. Pharmacological suppression of the PI3K-Akt pathway results in the nuclear aggregation of β-catenin and FOXO3a, which increases metastasis in colon carcinoma cells with aberrant canonical Wnt/β-catenin signaling [70]. These findings demonstrate that depending on the tissue type, canonical Wnt/β-catenin signaling can facilitate EMT either actively or passively. The strong concomitant expression of Wnt5a/b, Fzd2, and EMT landmarks suggests that non-canonical Wnt/β-catenin signaling is implicated in EMT. It has been demonstrated that Fyn and Stat3 activation by Fzd2 promotes EMT and cell migration. In a colon cancer xenograft mouse model, tumor development and metastasis were reduced when Fzd2 was targeted by a particular antibody [71].

Exosomes have recently been identified as a possible mechanism by which cancers establish their metastatic microenvironment [72]. Exosomes are tiny vesicles produced by cells and are used for signal transduction. It has been demonstrated that they can internalize β-catenin or serve as carriers of active Wnt ligands [73]. By activating the Wnt/PCP pathway, exosomes released by fibroblasts in the tumor microenvironment can augment the migration and protrusive behavior of breast cancer cells. In orthotopic mouse models, it has been shown that the co-injection of breast carcinoma cells with fibroblasts encourages metastasis. The coupling of Wnt11 to exosomes produced by fibroblasts is the mechanism by which this occurs [74].

Circulating tumor cells (CTCs) are another mechanism implicated in how distant metastasis spreads [75]. For prostate and pancreatic cancers, single-cell RNA sequencing of CTCs was executed, and these studies found a function for Wnt signaling. Wnt2 expression boosted anchorage-independent sphere generation and the potential of pancreatic CTCs to metastasize [76]. In a different study, it was discovered that prostate CTCs that are resistant to androgen receptor suppression have increased levels in the non-canonical Wnt signaling pathway [77]. Together, there is accumulating evidence that Wnt signaling can encourage cancer metastasis and angiogenesis in a tissue-specific manner.

### 2.3. Wnt Signaling in Tumor Immunity

The Wnt/β-catenin signaling pathway is implicated in tumor immune exclusion, in addition to its direct contribution to tumorigenesis [78,79]. Although research has suggested a link between CD8+ T-cell infiltration and APC alterations in colon cancer [4], the Cancer Genome Atlas (TCGA) has further demonstrated a more thorough link between T-cell infiltration and Wnt/β-catenin signaling. Immunohistochemical investigation of patient cohorts enrolled in the TCGA revealed that 13% of solid cancers had Wnt-linked alterations in genetic targets, such as Axin (1, 2), CTNNB1, and APC (1, 2), and that 80% of the studied solid cancers had stimulated Wnt/β-catenin signaling. These findings also demonstrated a correlation between poor CD8+ T-cell infiltration and elevated cytoplasmic β-catenin levels [80]. In malignancies and immunotherapies, T-cell infiltration is the key to self-destruction. Dendritic cells (DCs) typically recognize cancer cell antigens and stimulate B cells to generate antibodies. Moreover, DCs stimulate the development of naive T cells into cytotoxic T lymphocytes, which are then attracted to the tumor location and used to destroy cancer cells [81,82,83]. Increased expression of Wnt/β-catenin signaling aids in tumor evasion from immune surveillance, negates or resists chemotherapy, and increases susceptibility to recurrence [84,85]. The discovery of novel immunotherapies will be facilitated by focusing on the mechanisms through which Wnt/β-catenin signaling controls immune cells and immunologically mediated anticancer responses.

## 3. Flavonoids as Modulators of Dysregulated Wnt/β-Catenin Pathway in Cancer

**Figure 2:**
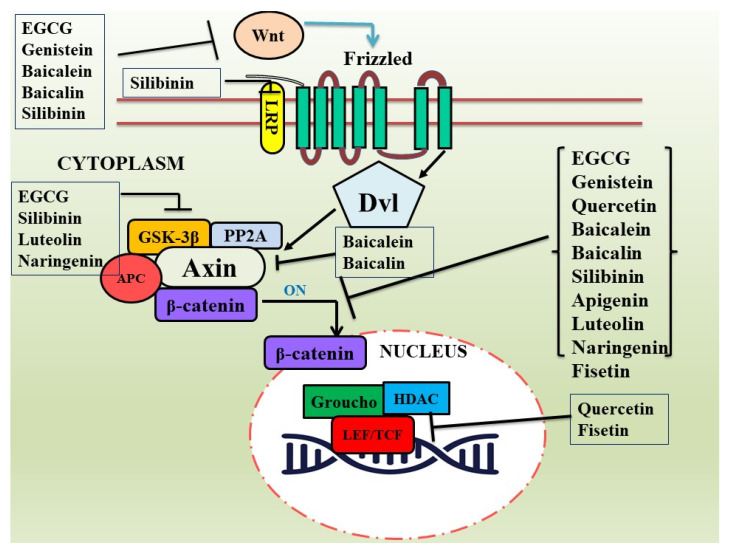
Effects of various flavonoids on the Wnt/β-catenin pathway. Flavonoids that modulate the Wnt cell signaling pathway by targeting Wnt ligands, receptors, intermediate signaling molecules, and downstream effector molecules in different cancer models are listed here.

Phytochemicals, particularly polyphenols, are among the most diverse and extensively researched classes of naturally occurring substances. This class includes subgroups of flavonoids and nonflavonoids. Flavonoids, also known as polyphenolic chemicals, are one of the most distinctive families of substances involved in plant metabolism and comprise an extensive group of natural compounds [86]. The subclasses, including flavones, chalcones, flavonols, and flavanones, are used to categorize almost 6000 of these compounds [87]. These substances eliminate the need to introduce foreign substances, which may cause individual complications. Because they are typically non-toxic, readily available, and more affordable than synthetic substances, they may result in the inhibition of various ailments in healthy individuals. Many studies have established the chemoprotective potential of flavonoids against various carcinomas [88]. It is generally known that a high consumption of fresh fruits and vegetables, particularly those rich in vitamins A, C, and E as well as beta-carotene, flavonoids, and other phytochemicals, protects against a variety of common malignancies in humans, including colon, breast, prostate, and lung cancers. Several substances have been proven to have antitumor properties in case–control investigations, cell culture experiments, and animal studies [89,90,91]. Recent research has shown that the ability of flavonoids to alter the Wnt/β-catenin signaling cascade is closely related to their antitumor properties [92,93]. Moreover, the ligand–receptor interaction (Wnt/Frizzled/LRP5/6) and methylation of targets encoding pathway elements, such as Wnt inhibitory factor 1 (WIF), have all been found to be affected by flavonoids [94,95]. Recent research reports have focused on the identification of numerous powerful compounds as potent inhibitors of the Wnt/β-catenin pathway, including EGCG (epigallocatechin-3-gallate), quercetin, genistein, kaempferol, baicalein, silibinin, naringenin, apigenin, fisetin, and luteolin (Figure 2) (Table 1). This section describes numerous distinct suppressing agents of the Wnt/β-catenin signaling pathway that have been identified in the analyses of these plant components.

### 3.1. Epigallocatechin-3-Gallate (EGCG)

Due to the existence of polyphenolic constituents in tea, several epidemiological research studies have demonstrated a potent reduction in the prevalence of carcinoma among those who regularly consume tea [96]. Green tea contains a lot of catechin, one of the most notable and well-known flavonoids. In a cup of brewed green tea, catechin EGC and epigallocatechin gallate (EGCG) make up between 30 and 40 percent of the dry weight (EGC). The most abundantly reported catechin in green tea is EGCG (epigallocatechin-3-monogallate). Although EGCG significantly inhibits the Wnt/β-catenin pathway, its mechanism still needs to be elaborated in detail. In a recent study, the Wnt signaling pathway was targeted, offering new information on how to prevent and treat gastric carcinoma. Reducing the expression levels of p-catenin (Ser552), β-catenin, p-GSK3 (S9), and EGCG blocked gastric cancer cell growth and showed that this suppressive action was correlated with canonical Wnt/β-catenin signaling [97].

Research on breast carcinoma has demonstrated that EGCG can block Wnt/β-catenin signaling without altering the expression levels of β-catenin by activating a transcriptional repressor called HBP-1. This has been demonstrated to be mediated by increased HBP1 mRNA stability [98,99]. Chen et al. further stated that EGCG treatment reduced the Wnt/β-catenin pathway activity, whereas LiCl-triggered activation of the pathway reversed the inhibitory potential of EGCG on spheroidal formation, cell growth, CSC markers, and death in colorectal cancer stem cells [100]. By inhibiting the Wnt/β-catenin pathway, EGCG also exerts an anticancer effect on lung CSCs in a similar manner [101].

It has been demonstrated that β-catenin protein (intracellular) stability is controlled by two APC-dependent mechanisms. The (APC/Axin/CK1/GSK-3β)-mediated pathway is the first pathway, followed by the (APC/Siah-1)-dependent pathway. Investigations further revealed a later mechanism that is not needed for EGCG-mediated β-catenin destruction [102]. According to Oh et al., treatment with EGCG did not downregulate mutant β-catenin at the Ser45 phosphorylation site of CK-1 or at the Ser37 phosphorylation site of GSK-3β. Phosphorylation of these special moieties is necessary for EGCG-induced β-catenin breakdown. Additionally, the authors found that the stability of β-catenin protein (intracellular) complex was unaffected by blocking GSK-3β activity or depleting it prior to EGCG treatment. They concluded that GSK-3 was not necessary for the EGCG-mediated degradation of β-catenin. Further research has established that EGCG can cause β-catenin degradation through a β-TrCp-mediated proteasomal mechanism, which in turn inhibits proliferation. EGCG seems to have greater efficacy than other catechins in preventing oxidative stress and tumorigenesis [102]. Studies have demonstrated that EGCG is a powerful Wnt inhibitor because it can reduce β-catenin levels and β-catenin/TCF-4 receptor activation in a dose-responsive manner [103]. Another effect of EGCG treatment on lung cancer H460 and A549 cell lines is a decrease in cytosolic β-catenin expression [104]. GSK3-α and GSK3-β activities has been demonstrated to be inhibited by EGCG treatment in HT29 colon cancer cells [105]. Additionally, it has been demonstrated that EGCG can block canonical Wnt signaling by downregulating the expression level of luciferase related to TCF/LEF [106]. In a study conducted by Singh et al., it was shown that EGCG can augment the amount of serine 33/37 residue in β-catenin via activating GK1/GSK-3. In skin cancer A431 and SCC13 cells, this promotes β-catenin breakdown and leads to an ensuing decrease in the nuclear aggregation of phosphorylated β-catenin [107]. It has been shown that EGCG administration reduces the expressions of DNMT1, Wnt, and β-catenin in the PC12 cell line, supporting the hypothesis that the Wnt/β-catenin signaling pathway is linked to cancer cell death [108]. The primary targets, GSK-3 and β-catenin, of the Wnt/β-catenin signaling pathway are downregulated by EGCG to produce its anticancer effects in human osteosarcoma cell lines MG63, 143B, and SaoS2 [109]. These results imply that EGCG may be a therapeutic candidate for the management of cancer by targeting abnormal Wnt/β-catenin signaling pathway.

### 3.2. Genistein

Genistein is a prominent isoflavone that is abundant in many plants, such as soybeans, tofu, and broccoli. Genistein, which has the chemical name [4′,5,7-trihydroxy isoflavonoid], can be found in food either in its free or esterified form. It has long been known that using soy products is associated with a lower chance of developing cancer. This is largely because soy products contain high genistein levels. This substance is found in *Genista tinctoria* L. plant and is soluble in different polar solvents. As previously mentioned, foodstuffs containing a soy base are the principal source of genistein [110]. Although genistein exhibits the desired bioavailability from a pharmacokinetic perspective, no proper safety assessment of genistein has yet been reported regarding its toxicokinetics. Studies have shown that isoflavones can significantly reduce β-catenin/Tcf-driven expression in AGS gastric carcinoma cells [111,112]. Moreover, Sarker et al. showed that isoflavones, particularly genistein, can increase GSK-3 expression, promote β-catenin binding to GSK-3, and increase β-catenin phosphorylation, which collectively inhibit the growth of prostate cancer [113]. Genistein can also attenuate Wnt-1-mediated cellular growth and its impact on c-Myc, VIZ, and cyclin D1 [114,115].

Studies have demonstrated that genistein inhibits Wnt signaling, which is linked to a decrease in pre-neoplastic lesions in the colon of male Sprague Dawley rats. Moreover, genistein administration suppresses the level of Wnt key elements, including Cyclin D1, c-Myc Wnt5a, Sfrp1, Sfrp2, and Sfrp5 [116,117]. Subsequent research has revealed that this phytochemical greatly reduces the level of β-catenin (CTNNBIP1) in colon cancer HT-29 cells [118].

Research using RT-PCR analysis has shown that genistein has anti-colorectal cancer properties mediated by the Wnt signaling pathway [119]. In SW1116 colon cancer cells, genistein lowered the level of WNT5a CpG island methylation, although DLD-1 and SW480 cells showed no such alteration. Additionally, genistein increased the expression of the sFRP2 gene by demethylating its silenced promoter in the colon cancer DLD-1 cell line, which inhibited β-catenin-mediated Wnt signaling [120]. In both in vitro and clinical RCC samples, miR-1260b was found to be highly expressed and dramatically reduced by genistein. Moreover, genistein decreased the expression of miR-1260b target genes, including sFRP1, Dkk2, and Smad4, thereby demonstrating a relationship with the Wnt signaling pathway [121]. By drastically reducing the mRNA levels of Wnt target genes, such c-myc and β-catenin in acute leukemia cells, genistein blocked the Wnt signaling pathway [122].

### 3.3. Quercetin

Quercetin, a plant flavonol derived from the polyphenol family, is a beneficial, readily available, and extremely potent natural chemical. It is abundant in fruits, vegetables, leaves, and other plants. Quercetin is employed to treat a wide range of ailments, such as malignancies, diabetes, and coronary heart diseases. Numerous studies have examined the antitumor effects of quercetin on cancer progression through signal transduction pathways, including PI3K/protein kinase B (AKT), Wnt/β-catenin, Janus kinase (JAK), signal transducer and transcription activator (STAT), NF-kB, and mitogen-activated protein kinase (MAPK) signaling cascades [123]. Numerous studies have shown that by targeting the Wnt/β-catenin pathway, the antitumor activity of quercetin has multidimensional effects. Mojsin et al. found that quercetin reduces β-catenin-dependent transcriptional efficacy in teratocarcinoma NT2/D1 cells by preventing SOX2, Nanog, and Oct4 mRNA levels, as well as inhibiting β-catenin nuclear movement [124]. Furthermore, Kim et al. demonstrated the antitumor efficacy of quercetin by inducing programmed cell death (apoptosis) in murine mammary carcinoma 4T1 cells. Recent research revealed that quercetin treatment resulted in enhanced expression of Wnt pathway regulators, including Dickkopf-related proteins (DKK) 1, 2, and 3, and concomitantly reduced cell viability [125]. Shan et al. examined Wnt signal transduction in human colon cancer SW480 cells, and they reported that quercetin reduced the level of cyclin D1 and survivin, two proteins that are associated with cell cycle regulation and death [126].

In a different study, Park et al. proposed that quercetin is a potent inhibitor of β-catenin/Tcf signaling in colon cancer SW480 cell lines and that decreased β-catenin/Tcf transcriptional ability is a result of reduced β-catenin (nuclear) and Tcf-4 proteins [127]. In a recent investigation using HT29 colon cancer cells, the effect of quercetin on a crucial regulator of the Wnt pathway, GSK3, was examined. Quercetin did not substantially prevent GSK3-α and GSK3-β at the selected doses; the total β-catenin expression in HT29 cells was almost unchanged. Thus, different biological and physiological conditions can result in various types of responses [105]. TGF-β is a key player in the metastasis and carcinogenesis of prostate carcinoma, and alterations in the elements of the Wnt signaling pathway are connected to different types of malignancies, including prostate cancer. In a study, quercetin demonstrated its anticancer effect in the prostate cancer PC-3 cell line through changes in EMT markers and Wnt signaling pathway components [128].

### 3.4. Baicalein and Baicalin

Baicalein (5,6,7-trihydroxyflavone) is a member of active flavonoids that is mostly present in the dried roots of the medicinal herb *Scutellaria baicalensis*. It has received a great deal of interest owing to its potential to inhibit cellular growth and apoptotic induction. Moreover, baicalein inhibits tumor growth by altering several cell signaling pathways, including p-Akt, p-mTOR, p-IB, and NF-kB [129]. In an osteosarcoma cell line, baicalein suppressed cell growth, boosted miR-25 expression, and controlled the Wnt/β-catenin pathway. Furthermore, baicalein and miR-25 enhanced GSK-3β expression and decreased Axin2 and β-catenin expressions. In addition, downregulation of miR-25 enhanced Axin2 and β-catenin expressions while decreasing GSK-3β expression [130]. Baicalein appeared to decrease overall β-catenin expression in osteosarcoma cells. It was observed that treatment with baicalein had no effect on the production of cytoplasmic β-catenin, which is transported from the cytoplasm to the nucleus and activates Wnt signaling. Moreover, baicalein treatment downregulated the expression levels of Wnt/β-catenin downstream effector genes, CD44, Oct3/4, and CCND (1,2), and survival. These findings imply that baicalein alters the translocation of the canonical Wnt/β-catenin pathway from the cytoplasm to the nucleus [131]. Osteocytes undergo carcinomatous transformation as a result of increased Wnt/β-catenin signaling, which aids in the growth of osteosarcomas. As determined by q-PCR and Western blotting, this mechanism is linked to lower expression of β-catenin and its crucial targets c-MYC. In line with this, subsequent research studies also revealed that baicalein targets several molecular markers through Wnt/β-catenin signaling to decrease osteosarcoma cell proliferation and promote cell death [132,133]. According to Xia et al., baicalein reduces the growth of cervical cancer cells by targeting the Wnt/β-catenin signaling pathway and CCND1. In cervical carcinoma HeLa, CaSki, C-33A, MS751, SN12C, and KBV1 cells, baicalein suppressed β-catenin nuclear movement and Wnt activity [134]. It has been demonstrated that baicalein can alter the mRNA and protein levels of β-catenin and its well-known downstream targets (cyclin D1, c-Myc, and Axin2) in T-lymphoblastic leukemia (T-ALL) [135]. Another study using breast cancer cells showed that baicalein exhibits antimetastatic properties by inhibiting SATB1 and the Wnt/β-catenin pathway. Baicalein significantly downregulates Wnt/β-catenin-targeted genes (Wnt1 and β-catenin) at the transcriptional and protein levels [136].

Furthermore, another important flavonoid of *Scutellaria baicalensis*, baicalin (7-D-Glucuronic acid-5,6-dihydroxyflavone), also demonstrates antitumor properties by targeting the Wnt/β-catenin signaling pathway in a few studies. In human osteosarcoma cell lines, baicalin has been demonstrated to activate apoptosis and autophagy by inhibiting the β-catenin signaling pathways [137]. Another study found that baicalin decreased the gene and protein expression levels of β-catenin in advanced-stage metastatic breast cancer cell lines. Upregulation of β-catenin by adenoviruses reversed these favorable impacts of baicalin on the migration and angiogenesis of breast cancer cells as well as their EMT [138].

### 3.5. Silibinin

Silibinin (flavonolignan) is a well-known natural dietary supplement extracted from milk thistle seed and has demonstrated biological activity against a range of malignancies via pleiotropic mechanisms [139]. Extensive molecular analysis indicated that silibinin targets signaling molecules responsible for the regulation of EMT, protease activation, migration, and invasion, as well as supporting tumor–microenvironment components, thereby preventing metastasis. Traditional uses of silibinin include dietary supplements for hepatoprotection; however, it has also been shown to have antitumor effects in a variety of in vitro and in vivo models of solid cancers, including carcinomas of the colon, skin, breast, lung, prostate, and kidney [140]. It has been established that its activity is related to regulation of the Wnt/β-catenin pathway. A human colorectal cancer cell line (SW480) and a xenograft model, where silibinin suppressed tumor progression by reducing the levels of β-catenin, c-Myc, and cyclin D1, also showed that the compound-induced reduction in cell growth was linked with the repression of the Wnt/β-catenin pathway [141]. In a different colon cancer animal study using A/J mice caused by AOM, silibinin administration had the same effect on tumor occurrence and multiplicity [142]. Comparable outcomes have also been observed in other in vivo models of colon tumorigenesis [143,144]. Additionally, in vitro experiments have demonstrated that silibinin prevents the motility and invasion of PC3 prostate cancer cells through a variety of mechanisms, including an increase in E-cadherin at the cell membrane and a decrease in nuclear β-catenin [145]. Another study showed that the Wnt co-receptor LRP6 is suppressed by silibinin and that its anticancer property is mediated by its influence on Wnt/LRP6 signaling in prostate and breast cancer cells [146]. An in vivo study using an ApcMin/transgenic mouse model of intestinal tumorigenesis further supported the anticancer efficacy of silibinin. This natural compound inhibits polyp growth in the small intestine and colon, and its anticarcinogenic efficacy is mediated by a reduction in β-catenin expression and transcriptional activity [147]. Additionally, Fan et al. showed that silibinin attenuates RCC metastasis and EMT in vitro and in vivo by modulating the Wnt/β-catenin signaling pathway. They also demonstrated that silibinin blocks the Wnt/β-catenin signaling cascade in an autophagy-mediated manner. The antimetastatic properties of silibinin against RCC are attributed to the autophagic destruction of β-catenin caused by silibinin [148].

### 3.6. Apigenin

Apigenin (4′,5,7,-trihydroxyflavone) is a natural flavonoid abundant in many fruits and vegetables.

The biological and pharmacological aspects of apigenin have been studied for many years. A growing body of studies have revealed that apigenin can modify the expression of important signaling pathways implicated in the carcinogenesis process, thereby inducing apoptosis [149]. Recent studies have shown that apigenin can reduce different kinds of malignancies, such as prostate, breast, lung, colorectal, liver, leukemia, ovarian, pancreatic, and cervical cancers. This is accomplished by inhibiting cancer cell metastasis, triggering apoptosis, and increasing immunity [150]. According to recent data, apigenin exposure has a directly impact on the Wnt/β-catenin expression [151]. The expression levels of downstream Wnt/β-catenin signaling effectors, including AXIN2, cyclin D1, and c-MYC, have also been shown to be modulated by apigenin [151]. Further research has revealed that apigenin significantly targets the crucial elements of Wnt/β-catenin signaling; however, its effects on LRP5 and Dishevelled (Dvl) are restricted [152]. In addition to inhibiting β-catenin movement to the nucleus through modulation of the PI3K/AkT/mTOR signaling pathway, apigenin decreases its accumulation and stability in the cytoplasm in a dose-responsive manner [152]. Moreover, apigenin overexpression inhibits the production of proto-oncogenes and suppresses the invasion and metastasis of colorectal cancer by suppressing Wnt/β-catenin signaling, while promoting the expression of E-cadherin and preventing the transportation of β-catenin to the nucleus [153]. These results suggest that apigenin may be a potential therapeutic alternative for the management of colorectal cancer.

According to Xu et al., apigenin reduced colorectal cancer cellular growth, migration, metastasis, and organoid development by impeding the Wnt/-catenin signaling pathway. Apigenin suppressed the stimulation of β-catenin/TCF/LEF signal by repressing the nuclear movement of β-catenin, which was increased by LiCl in a dose-responsive manner [154].

A long noncoding RNA, H19, which is typically increased in HCC, is known to play a critical role in promoting carcinogenesis and cancer development. Apigenin was found to downregulate H19 in a mouse model of xenograft tumors, which resulted in the attenuation of canonical Wnt/β-catenin signaling and tumor development [155]. Furthermore, Liu et al. demonstrated that apigenin reduced invasion and suppressed the proliferation of human OS cells by deactivating Wnt/β-catenin signaling. The repressive effect of apigenin on osteosarcoma cells was inversed by the upregulation of β-catenin, but it was strengthened by β-catenin downregulation [156]. In the dorsolateral prostate of TRAMP mice, apigenin treatment led to a higher expression of E-cadherin and lower expressions of β-catenin (nuclear), cyclin D1, and c-Myc. Similar outcomes were observed in TRAMP mice that already had tumors. Further similar results were observed when cancer cells were treated with β-catenin siRNA; apigenin exposure in DU145 prostate cancer cells increased E-cadherin protein expression, prevented nuclear movement of β-catenin and its accumulation in the cytoplasm, and declined c-Myc and cyclin D1 levels. These findings show that apigenin inhibits prostate tumorigenesis in TRAMP mice, at least in part, by preventing β-catenin signaling [157].

### 3.7. Luteolin

Luteolin (3′,4′,5,7-tetrahydroxyflavone) is a flavonoid that is present in different plants, medicinal herbs, fruits, and vegetables. It functions as an antitumor agent against different forms of human malignancies, such as glioblastoma, pancreatic, prostate, breast, and colon cancers. Moreover, it prevents the growth of cancer cells both in vitro and in vivo by preventing the proliferation of tumor cells, shielding them from carcinogenic stimuli, activating cell cycle arrest, and causing cell death via various signaling pathways [158]. In a 2013 study, it was found that luteolin administration in HCT-15 CRC cells had potent anti-proliferative effects by blocking Wnt/β-catenin signaling, triggering apoptotic cell death, and arresting the G2/M phase of cell growth [159]. It was also demonstrated that luteolin suppressed colon tumorigenesis induced by azoxymethane (AOM) by lowering the incidence and size of tumors. Luteolin inhibited cell proliferation by lowering the PCNA index and the number of argyrophilic nucleolar organizer region (AgNOR)/nuclei. This substance also prevents colon carcinogenesis by reducing AOM-induced cell proliferation through the participation of β-catenin, glycogen synthase kinase (GSK)-3, and cyclin D1, which are crucial elements in the Wnt signaling pathway [160]. According to Lin et al., luteolin also inhibited β-catenin mRNA and protein expression both in vitro and in vivo. They showed that luteolin significantly prevented breast cancer metastases by reversing EMT, which might be caused by β-catenin downregulation [161]. Han et al. revealed the efficacy of luteolin in prostate cancer PC-3 cells through the FZD6-mediated Wnt signaling pathway. It has also been shown that luteolin suppresses Wnt signaling irrespective of GSK-3β in prostate cancer cells by attenuating β-catenin transcriptional activity in GSK-3β-depleted cells [162].

### 3.8. Miscellaneous

Naringenin (4′,5,7-trihydroxyflavanone) is a prominent bioactive compound primarily found in citrus fruits, such grapefruits and other fruits, as well as in medicinal herbs. It belongs to the flavonoid class of polyphenols. As a herbal remedy, naringenin possesses significant pharmacological properties, including antioxidant, anti-inflammatory, neuroprotective, hepatoprotective, and anticancer activities, as per currently available reports. In vitro and in vivo investigations have demonstrated that carcinogens are rendered inactive after exposure to naringenin (pure), naringenin-loaded nanoparticles, or naringenin combined with chemotherapeutic drugs in a variety of malignancies. Naringenin suppresses the development of cancer through a variety of mechanisms, including pro-apoptosis, cell cycle arrest, inhibition of invasion, and modulation of several signaling pathways, including the Wnt/β-catenin, NF-kB, PI3K/Akt, and TGF-β pathways [163]. In gastric cancer cells, naringenin has also been shown by Lee et al. to suppress β-catenin/Tcf signaling through an unidentified mechanism [164]. Additional analysis revealed that 6-C-(E-phenylethenyl) naringenin (6-CEPN) has potent anti-liver cancer activity that is at least partially regulated by reducing the stemness of hepatocellular cells through a mechanism that involves Wnt/β-catenin signaling. It has been revealed that 6-CEPN inhibits nuclear translocation of β-catenin and causes its destruction by blocking Wnt/β-catenin signaling [165].

Fisetin (3,3′,4′,7-Tetrahydroxyflavone) has recently been identified as a Wnt/β-catenin signaling inhibitor [166]. Fisetin treatment of melanoma cells caused G1-phase arrest, reduced cell viability, and promoted disruption of Wnt/β-catenin signaling. The expression of Wnt protein and its co-receptors decreased along with this action, and endogenous Wnt inhibitor expression increased concurrently. Fisetin-treated cells displayed elevated cytosolic contents of Axin and β-TrCP and reduced GSK-3β phosphorylation in conjunction with reduced stability of β-catenin. Positively governed TCF targets, including c-myc, Mitf, and Brn-2, were downregulated as a consequence of fisetin-regulated interference with the enhanced cooperation among β-catenin and TCF-2 [166]. Interestingly, fisetin has been used to pharmacologically target Wnt/-catenin signaling dysregulation in colorectal cancer cells. When phosphorylated, β-catenin is ubiquitinated for destruction, whereas dephosphorylation causes stability and nuclear aggregation to transcriptionally modulate the transcription of target genes [167].

**Table 1:** Preclinical antitumor therapeutic interventions with different flavonoids targeting the Wnt/β-catenin signaling pathway in various cancer models.

Compound	Cancer Type	Cancer Model	Target of Wnt/β-Catenin Signaling Pathway	References
EGCG (C_22_H_18_O_11_)	Gastric cancer	SGC-7901	p-β-catenin and p-GSK3β	[97]
	Breast cancer	MDA-MB-231	GSK-3β, Wnt, and c-myc	[99]
	Colon cancer	DLD-1 and SW480	GSK-3β, β-catenin, and c-myc	[100]
	Lung cancer	A549 and H1299	GSK-3β and β-catenin	[101]
	Colon cancer	SW480 and HCT116	β-catenin, cyclin D1, and c-myc	[102]
	Colon cancer	HT-29	GSK3-α and-β, β-catenin	[105]
	Skin cancer	A431 and SCC13	Β-catenin, GSK-3β, and casein kinase1α	[107]
	Neuroendocrine tumor	PC12	β-catenin and Wnt-3a	[108]
	Osteosarcoma	MG63, 143B, and SaoS2	GSK-3β and β-catenin	[109]
Genistein (C_15_H_10_O_5_)	Colon pre-neoplasia	Sprague Dawley rats	Wnt5a, Sfrp1, Sfrp2, and Sfrp5	[116]
	Colon cancer	Sprague Dawley rats	Sfrp2, Sfrp5, and Wnt5a	[117]
	Colon cancer	HT-29	β-catenin	[118]
	Colon cancer	DLD-1	β-catenin	[119]
	Colon cancer	SW1116	WNT5a	[120]
	Renal cell carcinoma	Human sample (43)	sFRP1, Dkk2, and Smad4	[121]
	Renal cancer	A-498	sFRP1, Dkk2, and Smad4	[121]
	Acute leukemia	U937 and Jurkat	c-myc and β-catenin	[122]
Quercetin (C_15_H_10_O_7_)	Teratocarcinoma	NT2/D1	β-catenin	[124]
	Mammary cancer	4T1	β-catenin	[125]
	Colon cancer	SW480	β-catenin/Tcf	[126,127]
	Prostate cancer	PC-3	cyclin D1 and β-catenin	[128]
Baicalein (C_15_H_10_O_5_)	Osteosarcoma	143 B, MG63, and U2OS	β-catenin	[130,131]
	Osteosarcoma	MG63	β-catenin	[132]
	Osteosarcoma	MG-63	β-catenin, c-myc, cyclinD1, and survivin	[133]
	Cervical cancer	HeLa, CaSki, C-33A, MS751, SN12C, and KBV1	c-myc and β-catenin	[134]
	Leukemia	Jurkat cells	c-Myc, cyclin D1, Axin2, and β-catenin	[135]
	Breast cancer	MDA-MB-231	Wnt1 and β-catenin	[136]
Baicalin (C_21_H_18_O_11_)	Osteosarcoma	HOS, MG63, U2OS, and 143B	β-catenin	[137]
	Breast cancer	MDA-MB-231 and BALB/c mice	β-catenin	[138]
Silibinin (C_25_H_22_O_10_)	Colon cancer	SW480 and HCT116; athymic (nu/nu) male nude mice	c-Myc, cyclin D1, and β-catenin	[141]
	Colon cancer	A/J mice	β-catenin and pGSK-3β	[142]
	Colon cancer	male Wistar rats	β-catenin	[143,144]
	Prostate cancer	PC-3 and C4–2B	LRP6 and Wnt3A	[145]
	Prostate cancer	PC-3, DU-145	LRP6 and Wnt3A	[146]
	Breast cancer	MDA-MB-231 and T-47D	LRP6 and Wnt3A	[146]
	Renal cell carcinoma	786-O and ACHN; BALB/c male nude mice	Wnt3a, GSK3β, and β-catenin	[148]
Apigenin (C_15_H_10_O_5_)	Colon cancer	SW480 and HCT15; C57BL/6 mice	β-catenin	[154]
	Hepatocellular carcinoma	SMMC-7721 and HepG2	β-catenin	[155]
	Osteosarcoma	U2OS and MG63	β-catenin	[156]
	Prostate cancer	C57BL/TGN TRAMP mice, DU145	c-Myc, cyclin D1, and β-catenin	[157]
Luteolin (C_15_H_10_O_6_)	Colon cancer	HCT-15	GSK-3β, β-catenin, and c-myc	[159]
	Colon cancer	Azoxymethane (AOM)-induced mouse	β-catenin, GSK-3β, and cyclin D1	[160]
	Breast cancer	MDA-MB-231, BT5-49; Female nude mice	β-catenin	[161]
	Prostate cancer	PC-3, DU145	GSK-3β, cyclin D1, and c-myc	[162]
Naringenin (C_15_H_12_O_5_)	Gastric cancer	AGS	GSK-3β and β-catenin	[164]
	Hepatocellular carcinoma	Huh7 and Hep3B	GSK-3β and β-catenin	[165]
Fisetin (C_15_H_10_O_6_)	Colon cancer	HCT116 and HT29	TCF4 and β-catenin	[166]
	Melanoma	Mel 928, WM35, and 451Lu	β-catenin	[167]

## 4. Limitations Associated with Natural Products as Anticancer Therapeutics

Natural bioactive substances obtained from plants have been used to treat various human health problems for a long time. Currently, they are a key source of drug discovery for the creation of contemporary medications. Despite the intriguing biological properties of many natural compounds, most of them cannot be used efficiently in therapeutic medications because of their intrinsic flaws, including low solubility, structural instability, short half-lives, poor bioavailability, and non-specific organ delivery. Furthermore, the use of these phytochemicals is further constrained by their poor pharmacokinetic profile, which includes rapid metabolism and short half-life in the body. To enhance their pharmacokinetic profile, derivatization of these phytochemicals is necessary. An ideal derivative is one that is comparable to or more effective than the original prototype in terms of solubility, bioavailability, tumor invasion, and dispersion. The development of new delivery methods, including nanotechnology, has led to several encouraging improvements in the pharmacokinetics of pharmaceuticals with limited solubility and low bioavailability and, in the case of phytochemicals, can dramatically overcome pharmacokinetic limitations. Table 2 provides a brief summary of the pharmacokinetic characteristics of the flavonoids described in this study [168].

## 5. Conclusions

In conclusion, the Wnt/β-catenin signaling pathway is crucial for cancer growth and progression. Wnt communicates extensively with other signaling pathways, such as the NF-κB, JAK/STAT, and Notch pathways, which provide cancer cells with a survival advantage, thereby making it crucial to target this pathway. Moreover, Wnt/β-catenin activation is crucial for maintaining CSCs that are highly tumorigenic and treatment-resistant and contribute to the survival and recurrence of tumors. Many natural compounds have been discovered to be Wnt/β-catenin signaling modulators over the past 10 years, and the majority of research studies have focused on examining the effectiveness of these modulators as cancer prevention and/or treatment agents. Further research on the impact of flavonoids, either alone or in conjunction with chemotherapeutics, on the modulation of Wnt/β-catenin signaling will advance our knowledge of this signaling cascade and enhance tumor prevention and treatment.

## Figures and Tables

**Table 2:** Prediction of the main pharmacokinetic parameters for selected anticancer flavonoids using the ADMETlab 2.0.

Compound	Properties	Parameters	Values
EGCG	Absorption	Caco-2 permeability	−6.306
		HIA	0.274
	Distribution	PPB	91.15%
		BBB penetration	0.019
	Metabolism	CYP1A2 inhibitor	0.095
		CYP2C19 inhibitor	0.124
		CYP2C9 inhibitor	0.174
		CYP2D6 inhibitor	0.037
		CYP3A4 inhibitor	0.142
	Excretion	CL	17.081 mL/min/kg
		T ½	0.87
	Toxicity	H-HT	0.092
		DILI	0.172
		FDAMDD	0.17
		Respiratory toxicity	0.085
Genistein	Absorption	Caco-2 permeability	−4.764
		HIA	0.01
	Distribution	PPB	97.55%
		BBB penetration	0.02
	Metabolism	CYP1A2 inhibitor	0.981
		CYP2C19 inhibitor	0.59
		CYP2C9 inhibitor	0.615
		CYP2D6 inhibitor	0.863
		CYP3A4 inhibitor	0.716
	Excretion	CL	7.8444 mL/min/kg
		T ½	0.876
	Toxicity	H-HT	0.092
		DILI	0.51
		FDAMDD	0.201
		Respiratory toxicity	0.087
Quercetin	Absorption	Caco-2 permeability	−5.204
		HIA	0.014
	Distribution	PPB	95.49%
		BBB penetration	0.008
	Metabolism	CYP1A2 inhibitor	0.943
		CYP2C19 inhibitor	0.053
		CYP2C9 inhibitor	0.598
		CYP2D6 inhibitor	0.411
		CYP3A4 inhibitor	0.348
	Excretion	CL	8.284
		T ½	0.929
	Toxicity	H-HT	0.1
		DILI	0.98
		FDAMDD	0.31
		Respiratory toxicity	0.072
Baicalein	Absorption	Caco-2 permeability	−4.981
		HIA	0.018
	Distribution	PPB	98.99%
		BBB penetration	0.013
	Metabolism	CYP1A2 inhibitor	0.971
		CYP2C19 inhibitor	0.144
		CYP2C9 inhibitor	0.644
		CYP2D6 inhibitor	0.638
		CYP3A4 inhibitor	0.183
	Excretion	CL	4.082
		T ½	0.881
	Toxicity	H-HT	0.084
		DILI	0.958
		FDAMDD	0.084
		Respiratory toxicity	0.332
Baicalin	Absorption	Caco-2 permeability	−6.34
		HIA	0.793
	Distribution	PPB	83.35%
		BBB penetration	0.05
	Metabolism	CYP1A2 inhibitor	0.037
		CYP2C19 inhibitor	0.017
		CYP2C9 inhibitor	0.005
		CYP2D6 inhibitor	0.01
		CYP3A4 inhibitor	0.004
	Excretion	CL	1.0
		T ½	0.855
	Toxicity	H-HT	0.178
		DILI	0.977
		FDAMDD	0.005
		Respiratory toxicity	0.046
Silibinin	Absorption	Caco-2 permeability	−6.255
		HIA	0.366
	Distribution	PPB	96.65%
		BBB penetration	0.024
	Metabolism	CYP1A2 inhibitor	0.038
		CYP2C19 inhibitor	0.12
		CYP2C9 inhibitor	0.664
		CYP2D6 inhibitor	0.31
		CYP3A4 inhibitor	0.785
	Excretion	CL	5.144
		T ½	0.274
	Toxicity	H-HT	0.079
		DILI	0.921
		FDAMDD	0.035
		Respiratory toxicity	0.027
Apigenin	Absorption	Caco-2 permeability	−4.847
		HIA	0.015
	Distribution	PPB	97.25%
		BBB penetration	0.012
	Metabolism	CYP1A2 inhibitor	0.988
		CYP2C19 inhibitor	0.588
		CYP2C9 inhibitor	0.602
		CYP2D6 inhibitor	0.792
		CYP3A4 inhibitor	0.833
	Excretion	CL	7.022
		T ½	0.856
	Toxicity	H-HT	0.072
		DILI	0.854
		FDAMDD	0.433
		Respiratory toxicity	0.266
Luteolin	Absorption	Caco-2 permeability	−5.208
		HIA	0.047
	Distribution	PPB	95.43%
		BBB penetration	0.009
	Metabolism	CYP1A2 inhibitor	0.981
		CYP2C19 inhibitor	0.124
		CYP2C9 inhibitor	0.576
		CYP2D6 inhibitor	0.559
		CYP3A4 inhibitor	0.549
	Excretion	CL	8.146
		T ½	0.898
	Toxicity	H-HT	0.084
		DILI	0.905
		FDAMDD	0.741
		Respiratory toxicity	0.22
Naringenin	Absorption	Caco-2 permeability	−4.803
		HIA	0.018
	Distribution	PPB	93.76%
		BBB penetration	0.042
	Metabolism	CYP1A2 inhibitor	0.917
		CYP2C19 inhibitor	0.793
		CYP2C9 inhibitor	0.823
		CYP2D6 inhibitor	0.745
		CYP3A4 inhibitor	0.855
	Excretion	CL	17.388
		T ½	0.774
	Toxicity	H-HT	0.098
		DILI	0.853
		FDAMDD	0.177
		Respiratory toxicity	0.34
Fisetin	Absorption	Caco-2 permeability	−4.987
		HIA	0.009
	Distribution	PPB	97.04%
		BBB penetration	0.009
	Metabolism	CYP1A2 inhibitor	0.95
		CYP2C19 inhibitor	0.097
		CYP2C9 inhibitor	0.535
		CYP2D6 inhibitor	0.532
		CYP3A4 inhibitor	0.62
	Excretion	CL	8.273
		T ½	0.92
	Toxicity	H-HT	0.127
		DILI	0.978
		FDAMDD	0.259
		Respiratory toxicity	0.074

**Abbreviations:** HIA: human intestinal absorption; PPB: plasma protein binding; BBB penetration: blood–brain barrier penetration; CL: clearance; T ½: half-life; H-HT: human hepatotoxicity; DILI: drug-induced liver injury; FDAMDD: maximum recommended daily dose. Data extracted from https://admetmesh.scbdd.com/ (accessed on 5 February 2023).

## Data Availability

Data sharing is not applicable to this article as no new data were created or analyzed in this study.
